# Successful Heart Transplantation Without Blood Transfusion in a Jehovah's Witness Patient With Congenitally Corrected Transposition of the Great Arteries: A Case Report

**DOI:** 10.7759/cureus.56997

**Published:** 2024-03-26

**Authors:** Yusuke Tsukioka, Valluvan Jeevanandam

**Affiliations:** 1 Cardiothoracic Surgery, University of Chicago Medicine, Chicago, USA; 2 Cardiac Surgery, University of Chicago Medicine, Chicago, USA

**Keywords:** right ventricular dysfunction, bloodless surgery, jehovah's witness, heart transplantation, congenitally corrected transposition of the great arteries (cctga)

## Abstract

We report a case of heart transplantation in a 68-year-old Jehovah's Witness patient with congenitally corrected transposition of the great arteries (ccTGA) who developed heart failure due to right ventricular dysfunction. The patient underwent successful heart transplantation without the use of blood products, employing meticulous hemostasis and careful surgical planning. This case highlights the anatomical considerations and challenges in transplanting a heart in a patient with ccTGA, including the reversed positions of the pulmonary artery and the ascending aorta. It also emphasizes the importance of tailored surgical strategies to achieve hemostasis and avoid blood transfusion in Jehovah's Witness patients. This case adds to the limited literature on heart transplantation in patients with ccTGA and demonstrates that heart transplantation can be successfully performed without blood transfusion in Jehovah's Witness patients.

## Introduction

Congenitally corrected transposition of the great arteries (ccTGA) is characterized by an inversion of the ventricles, with the right atrium connecting to the anatomically left ventricle (on the right side) leading to the pulmonary artery, and the left atrium connecting to the anatomically right ventricle (on the left side) giving rise to the aorta. Despite this inversion, the flow of blood remains similar to that in a normal heart, with venous blood directed to the pulmonary artery and arterial blood flowing to the aorta. However, ccTGA is frequently associated with complications such as ventricular septal defect (VSD) or VSD combined with pulmonary stenosis, each presenting distinct hemodynamics and clinical symptoms [1]. Due to the unique atrioventricular (AV) connections, arrhythmias such as AV block and episodes of tachycardia are common [2]. Furthermore, the anatomically right ventricle, unlike the anatomically left ventricle, is not capable of sustaining pressures above 120 mmHg over a lifetime, leading to the development of right ventricular failure in adulthood [3,4]. We present a heart transplantation performed on a Jehovah's Witness patient in their late 60s with ccTGA who developed heart failure due to right ventricular dysfunction, without complications of VSD, pulmonary stenosis, or AV block. We share the critical aspects of the surgery, illustrated with imaging findings.

## Case presentation

The patient, a 68-year-old Jehovah's Witness, presented with ccTGA. He was transferred from an external hospital due to exertional dyspnea, manifesting as shortness of breath upon ascending a single flight of stairs, classified as New York Heart Association (NYHA) functional class III. The patient's clinical presentation was further complicated by severe dysfunction of the systemic ventricle (anatomically a morphological right ventricle, RV), moderate-to-severe regurgitation of the systemic AV valve (morphologically a tricuspid valve, TV), and was on warfarin due to episodes of paroxysmal atrial fibrillation (AF). Notably, the patient had no prior surgical interventions for his ccTGA. For hemodynamic support, he was transferred to our facility with an intra-aortic balloon pump in place.

Preoperative examination

The laboratory findings show a hemoglobin level of 15.9 g/dL (Table 1).

**Table 1:** Laboratory findings

Parameter	Value	Reference Values
White Blood Cell Count (WBC)	4.9 × 10³/μL	4.5-11.0 × 10³/μL
Red Blood Cell Count (RBC)	4.60 × 10⁶/μL	4.5-5.9 × 10⁶/μL
Hemoglobin	15.9 g/dL	13.8-17.2 g/dL
Platelet Count	134 × 10³/μL	150-450 × 10³/μL
International Normalized Ratio (INR)	1.3	0.8-1.2
Creatinine	1.17 mg/dL	0.7-1.2 mg/dL
Estimated Glomerular Filtration Rate (eGFR)	68 mL/min/1.73 m²	>90 mL/min/1.73 m²

Transthoracic echocardiogram

The transthoracic echocardiogram reveals ccTGA. The systemic ventricle, which is morphologically a right ventricle, is found to be moderately dilated with severely reduced function, as indicated by an ejection fraction (EF) of 25%. Conversely, the pulmonary ventricle, morphologically a left ventricle, exhibits mild dilation and mild functional impairment. The systemic AV valve demonstrates bileaflet tethering and appears to have malcoaptation, leading to severe regurgitation. The left atrial volume index is elevated at 88.6 ml/m², suggesting left atrial enlargement. Additionally, a dilated inferior vena cava is observed, implying increased right atrial pressure. The aortic valve shows no significant stenosis, although trace regurgitation is present. Similarly, the pulmonic valve does not exhibit significant stenosis, with only trace regurgitation noted.

Electrocardiogram (ECG) 

The ECG demonstrates sinus bradycardia with a heart rate of 55 beats per minute. 

Computed tomography (CT) scan

**Figure 1:**
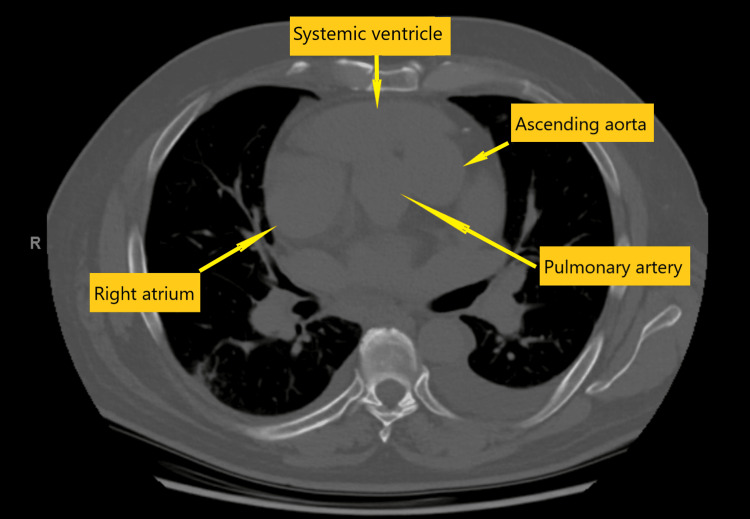
Preoperative CT scan The ascending aorta, arising from the morphological right ventricle (systemic ventricle), is positioned to the left of the pulmonary artery.

The CT scan reveals transposition of the great vessels, accompanied by mild dilation of the main pulmonary artery. The heart is noted to be enlarged. Additionally, a small pericardial effusion is present. The branching pattern of the arch vessels is characterized as bovine, indicating a common trunk for the innominate and left common carotid arteries. All of the arch branch vessels appear to be widely patent in their proximal portions, showing no signs of significant obstruction (Video 1 and Figure 1).

**Video 1:** Preoperative CT scan The ascending aorta, arising from the morphological right ventricle (systemic ventricle), is positioned to the left of the pulmonary artery in ccTGA. ccTGA: Congenitally corrected transposition of the great arteries

The preoperative right heart catheterization study indicates elevated pulmonary artery pressure and elevated left ventricular (LV) filling pressure (Table 2). 

**Table 2:** Preoperative right heart catheterization study

Parameter	Value	Reference Values
Right Atrium (RA)	14 mmHg	0-8 mmHg
Pulmonary Ventricle	100/17 mmHg	15-30/3-8 mmHg
Pulmonary Artery (PA)	92/36 (57) mmHg	15-30/5-15 mmHg
Pulmonary Capillary Wedge Pressure (PCWP)	32 mmHg	4-12 mmHg
PA Saturation (Sat)	72.30%	95-98%
SpO_2_	97%	95-100%
Blood Pressure (BP)	107/66 (82) mmHg	90-120/60-80 mmHg
Fick Cardiac Output (CO)	5.22 L/min	4-8 L/min
Fick Cardiac Index (CI)	2.57 L/min/m²	2.5-4.0 L/min/m²
Systolic Vascular Resistance (SVR)	1042 dynes・sec・cm⁻⁵	900-1400 dynes・sec・cm⁻⁵
Pulmonary Vascular Resistance (PVR)	4.78 Wood units	<2.0 Wood units

Intraoperative findings

After median sternotomy, the patient was placed on cardiopulmonary bypass (CPB) with cannulation of the transverse arch under the innominate vein using a 20 French (Fr) aortic cannula and bicaval cannulation (superior vena cava (SVC) 22 Fr venous cannula and inferior vena cava (IVC) 31 Fr venous cannula). The patient was cooled to 32°C, and pressure was temporarily reduced to 50mmHg with the aorta cross-clamped.

**Figure 2:**
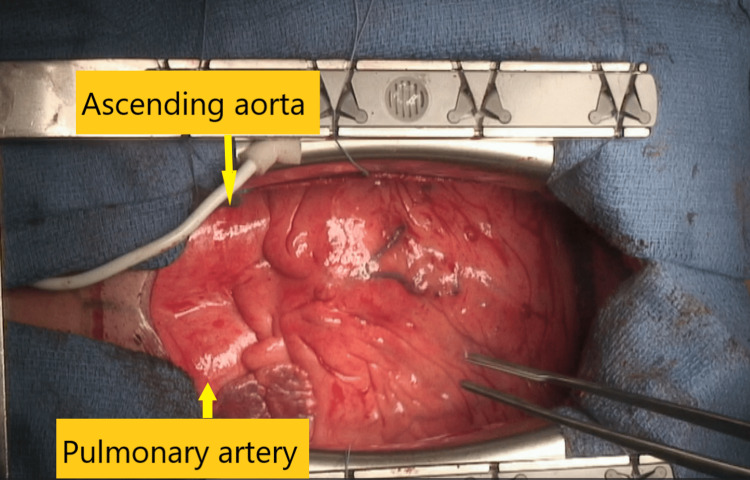
Intraoperative image The ascending aorta, arising from the morphological right ventricle (systemic ventricle), was positioned to the left of the pulmonary artery.

A transverse incision was made in the aorta, and the heart was explanted by transecting the pulmonary artery and the aorta. The positions of the pulmonary artery and the ascending aorta were reversed, with the ascending aorta located to the left of the pulmonary artery (Figure 2). Therefore, it was considered necessary to have a long pulmonary artery for the anastomosis. Accordingly, the recipient's pulmonary artery was sufficiently dissected from the ascending aorta and transected near the pulmonary valve. The recipient's ascending aorta was also sufficiently dissected from the surrounding tissue and transected near the aortic valve. The donor's pulmonary artery and aorta were harvested longer than usual. The right atrium was removed, and the pulmonary veins, IVC, and SVC were left for anastomosis.

**Figure 3:**
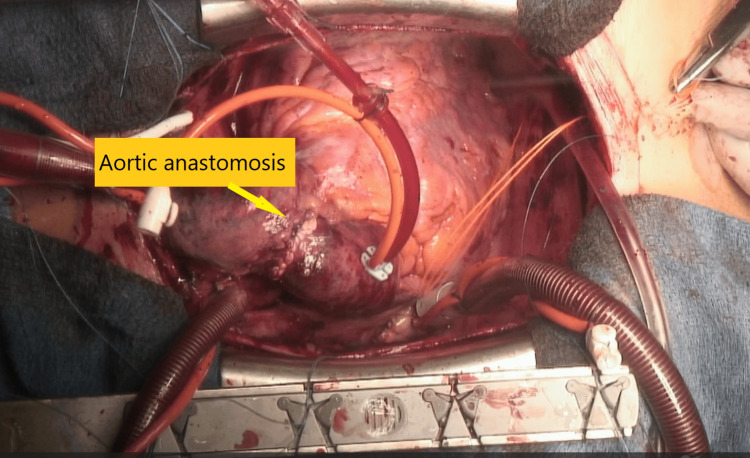
Image after heart implantation The ascending aorta was anastomosed longer than usual and shaped into an arch toward the ceiling to ensure that the pulmonary artery was not compressed or stenosed by the ascending aorta.

The donor's heart was prepared for transplantation by separating the PA, aorta, SVC, and pulmonary veins. A DeVega tricuspid annuloplasty was done with pledgeted 2-0 SurgiPro (Covidien, Mansfield, MA, USA). Left and right pulmonary venous anastomoses were performed with 3-0 SurgiPro. The IVC and SVC anastomoses (4-0 SurgiPro) followed. The pulmonary artery and aortic anastomosis (4-0 Surgipro) followed. The long pulmonary artery was anastomosed in a direction toward the right side of the body, taking care not to cause kinking. This anastomosis required careful attention as it would be difficult to achieve hemostasis later. Additionally, the ascending aorta was anastomosed longer to create space for the pulmonary artery to pass underneath it. By trimming the dorsal side of the ascending aorta shorter and the ventral side longer, the ascending aorta was designed to form a slight arch towards the ceiling (Figure 3). After deairing and weaning from CPB, decannulation and protamine administration followed. Hemostasis was easy and adequate. No blood products were administered.

Postoperative course

The postoperative course was uneventful, and hemodynamics remained stable. Hemoglobin levels consistently exceeded 11 g/dL, and no blood transfusion was required postoperatively (Table 3). The postoperative right heart catheterization study demonstrates significant improvement in pulmonary artery pressure, pulmonary capillary wedge pressure, and cardiac output (Table 4). Rehabilitation progressed smoothly, leading to discharge as planned.

**Table 3:** Postoperative laboratory findings (two weeks postoperative)

Parameter	Value
Hemoglobin	12.6 g/dL
Platelet Count	123×10³/μL

**Table 4:** Postoperative right heart catheterization study

Parameter	Value	Reference Values
Right Atrium (RA)	15 mmHg	0-8 mmHg
Right Ventricle (RV)	44/15 mmHg	15-30/3-8 mmHg
Pulmonary Artery (PA)	47/23 (32) mmHg	15-30/5-15 mmHg
Pulmonary Capillary Wedge Pressure (PCWP)	20 mmHg	4-12 mmHg
PA Saturation (Sat)	75.60%	95-98%
SpO_2_	95%	95-100%
Blood Pressure (BP)	135/63 (90) mmHg	90-120/60-80 mmHg
Fick Cardiac Output (CO)	7.8 L/min	4-8 L/min
Fick Cardiac Index (CI)	4.0 L/min/m²	2.5-4.0 L/min/m²
Systolic Vascular Resistance (SVR)	769 dynes・sec・cm⁻⁵	900-1400 dynes・sec・cm⁻⁵
Pulmonary Vascular Resistance (PVR)	1.5 Wood units	<2.0 Wood units

Echocardiogram

The echocardiogram reveals normal LV systolic performance with an EF of 55%. There are no significant abnormalities observed in the cardiac valves. Additionally, there is no evidence of stenosis in the ascending aorta or the pulmonary artery.

## Discussion

This case report describes heart transplantation in a 68-year-old Jehovah's Witness patient with ccTGA. While there are limited case reports of heart transplantation for ccTGA, to our knowledge, this is the first report of a bloodless surgery in a Jehovah's Witness patient with this condition [5-9]. We herein share our approach to performing bloodless heart transplantation in a patient with ccTGA, which is illustrated with images.

ccTGA is a rare congenital heart disease, constituting 0.5-1% of all cardiac anomalies. It is estimated that 5,000-10,000 individuals in the United States are affected by this condition [10]. Common complications associated with ccTGA include VSD, pulmonary stenosis, and AV block, with occurrence rates exceeding 70%, 40%, and 30% in adults, respectively [2]. In this case, the patient had ccTGA but did not exhibit these complications. The primary causes of heart failure in this patient were AV valve regurgitation, systemic right ventricular dysfunction, and AF.

A critical anatomical consideration in this heart transplantation was the reversed positions of the pulmonary artery and the ascending aorta. As previously mentioned, both the ascending aorta and the pulmonary artery were harvested longer in the recipient and the donor. During implantation, the anastomosis was performed such that the ascending aorta formed an arch, allowing the pulmonary artery to pass underneath it. This anastomosis was facilitated by lengthening the ascending aorta and the pulmonary artery, contrasting with the approach by Sue et al., who performed the anastomosis with the heart rotated clockwise [5]. In our case, the heart was also positioned with a slight clockwise rotation.

In surgeries for Jehovah's Witness patients, we proactively address coagulation issues by administering Vitamin K to correct the prothrombin time-international normalized ratio once a donor's heart becomes available for transplantation. We also emphasize the importance of achieving hemostasis of the sternum at the time of sternotomy. We meticulously achieve hemostasis of the bone marrow using Hemasorb (Abyrx, Irvington, NY, USA) and repeatedly cauterize the edges of the sternum at least three times during sternotomy with an electrocautery device. After administering heparin, we minimize the use of the cell saver to prevent the removal of platelets and coagulation factors. Additionally, we restrict the use of gauze during surgery, as excessive gauze can lead to the loss of blood components adhering to it. The gauze used is periodically washed and wrung out with saline as needed, and the blood washed from the gauze is either processed with a cell saver or returned to the cardiopulmonary bypass for reuse.

In conclusion, ccTGA is a rare condition with distinctive anatomical features. Nevertheless, with careful planning and execution, heart transplantation can be successfully performed without the need for blood transfusion, even in Jehovah's Witness patients.

## Conclusions

This case report underscores the feasibility of performing heart transplantation in a Jehovah's Witness patient with ccTGA. By utilizing a meticulous surgical approach and careful hemostasis, successful transplantation was achieved without the need for blood transfusion. This case adds to the limited literature on heart transplantation in patients with ccTGA and highlights the importance of tailored surgical strategies in managing complex congenital heart diseases in Jehovah's Witness patients.
